# Renal complications in patients with chronic hypoparathyroidism on conventional therapy: a systematic literature review: Renal disease in chronic hypoparathyroidism

**DOI:** 10.1007/s11154-020-09613-1

**Published:** 2021-02-18

**Authors:** Elvira O. Gosmanova, Pascal Houillier, Lars Rejnmark, Claudio Marelli, John P. Bilezikian

**Affiliations:** 1 Albany Medical College, Albany, NY USA; 2 Centre de Recherche des Cordeliers, INSERM, Sorbonne Université, Université de Paris, Assistance Publique-Hôpitaux de Paris, Paris, France; 3 Aarhus University and Aarhus University Hospital, Aarhus, Denmark; 4 Takeda Pharmaceuticals International AG, Zurich, Switzerland; 5 Division of Endocrinology, College of Physicians and Surgeons, Columbia University, 630 W 168th Street, Room 864, New York, NY 10032 USA

**Keywords:** Hypoparathyroidism, Chronic kidney disease, Nephrocalcinosis, Nephrolithiasis

## Abstract

A systematic literature review was performed to summarize the frequency and nature of renal complications in patients with chronic hypoparathyroidism managed with conventional therapy. Methodology was consistent with the recommendations outlined in the Preferred Reporting Items for Systematic Reviews and Meta-Analyses statement. Peer-reviewed journal articles with specified medical subject heading terms were identified using the PubMed, EMBASE, and Cochrane databases. Data were extracted from eligible articles based on prespecified parameters for clinical outcomes of renal calcifications and disease. Because of the heterogeneity of the data, a meta-analysis could not be conducted. From 1200 potentially relevant articles, data were extracted from 13 manuscripts that reported data for ≥1 of the 19 predefined renal outcomes for ≥10 adult patients (*n* = 11 manuscripts) or pediatric patients (*n* = 2 manuscripts). The collective data provide evidence that adult and pediatric patients with chronic hypoparathyroidism and treated with conventional therapy (oral calcium and active vitamin D) had an increased risk of renal complications. The reported rate of nephrolithiasis was up to 36%, with the lowest rates in studies reporting shorter duration of disease. The rate of nephrocalcinosis was up to 38%. Some studies reported a combined nephrolithiasis/nephrocalcinosis outcome of 19% to 31%. Data for renal disease that encompassed a range of renal insufficiency to chronic kidney disease were reported in 10 articles; the reported rates ranged from 2.5% to 41%. In patients who receive long-term treatment with oral calcium and active vitamin D, chronic hypoparathyroidism may be associated with an increased risk of renal complications compared with the general population.

## Introduction

Hypoparathyroidism is a rare endocrine disorder caused by absent or inappropriately low levels of parathyroid hormone (PTH) [1]. Mineral homeostasis cannot be maintained because of the loss of the PTH-controlled pathways involving bone, kidney and the gastrointestinal (GI) tract. The absorption of calcium in the GI tract is greatly decreased because of the loss of activation of 25-hydroxyvitamin D (25[OH]D) to 1,25-dihydroxyvitamin D (1,25 [OH]_2_D), which stimulates absorption of both calcium and phosphate [2]. The skeleton ceases to be a ready source of calcium because of exceedingly low bone turnover [3]. There is reduced calcium reabsorption and urinary phosphate excretion in the kidney because of the loss of the effect of PTH [2, 4]. The results of these pathophysiological factors in hypoparathyroidism are hypocalcemia and hyperphosphatemia [1, 2, 5].

Conventional treatment in patients with chronic hypoparathyroidism is oral calcium and active vitamin D (eg, calcitriol), as well as parenteral forms of vitamin D and thiazide diuretics as needed [1, 4, 5]. Over the lifetime of an individual, chronic hypoparathyroidism and long-term therapy with oral calcium and active vitamin D appear to be associated with an increase in the risk of renal complications based on a number of retrospective studies in adult or pediatric patients with chronic hypoparathyroidism [6–10]. A case-controlled retrospective study found that increased calcium-phosphate product (ie, [serum calcium concentration] × [serum phosphate concentration]) was associated with increased risk of renal disease in patients with hypoparathyroidism [8]. Mitchell et al. suggested that conventional treatment may increase the risk of hypercalciuria, itself a risk factor for nephrolithiasis, nephrocalcinosis, and impaired renal function [9].

The objective of this systematic literature review is to summarize the reported frequency and nature of renal complications in patients with chronic hypoparathyroidism managed conventionally with calcium and active vitamin D. The specific renal outcomes investigated were nephrolithiasis/kidney stones, nephrocalcinosis, and chronic kidney disease (CKD). In addition, estimated glomerular filtration rate (eGFR) levels were also investigated. Any reported associations between each of the renal outcomes and relevant biochemical or disease parameters in the published selected articles are included in this review.

## Systematic literature search

### Data sources

Methodology was consistent with the recommendations outlined in the Preferred Reporting Items for Systematic Reviews and Meta-Analyses (PRISMA) statement [11]. A methodology protocol specified the process. Primary eligibility criteria for the inclusion of peer-reviewed journal articles are listed in Table 1. Searches were conducted in PubMed^®^/MEDLINE^®^ for English-language abstract-containing articles published in peer-reviewed journals from database inception to 15 November 2018. Additional peer-reviewed journal articles not in PubMed^®^/MEDLINE^®^ were identified by similar searches conducted in EMBASE^®^ and Cochrane^®^ databases for the same timeframe.

**Table 1:** Primary Eligibility Criteria for Relevant Peer-Reviewed Journal Articles

Inclusion criteria	
Patient population	Adults, children, infants with hypoparathyroidism
Other population	Preclinical (hypoparathyroidism relevant)
Language	English language
Exclusion criteria	
Treatment interventions	PTH, PTH analogs, rhPTH(1–84), rhPTH(1–34)
Type of article	Review

PTH, parathyroid hormone; rhPTH, recombinant human parathyroid hormone

### Search strategy

The strategy employed a database search string composed of free text and controlled vocabulary terms (ie, medical subject headings [MeSH] for PubMed^®^/MEDLINE^®^). Selected MeSH terms included nephrocalcinosis, nephrolithiasis, kidney calculi, and all related terms for kidney stones, renal insufficiency, and chronic kidney/renal disease. This approach was broad based but also included precise terminology to capture publications that would potentially have data values for the predefined relevant clinical outcomes (Table 2). The following is the search string that was used, formatted for PubMed^®^/MEDLINE^®^: “Hypoparathyroidism”[Title] OR ((Hypoparathyroidism[MESH] AND kidney diseases[MESH] AND kidney[MESH]) OR (Hypoparathyroidism[MESH] AND hypercalciuria[MESH]) OR (Hypoparathyroidism[MESH] AND morbidity[MESH]) OR (hypoparathyroidism[MESH] AND hypercalcemia[MESH]) OR (hypoparathyroidism[MESH] AND hyperphosphatemia[MESH])) NOT (Hyperparathyroidism [MESH] OR adynamic bone disease[MESH]) AND ((Clinical Study[ptyp] OR Clinical Trial[ptyp] OR Clinical Trial, Phase I[ptyp] OR Clinical Trial, Phase II[ptyp] OR Clinical Trial, Phase III[ptyp] OR Clinical Trial, Phase IV[ptyp] OR Comparative Study[ptyp] OR Controlled Clinical Trial[ptyp] OR Dataset[ptyp] OR Meta-Analysis[ptyp] OR Observational Study[ptyp] OR Pragmatic Clinical Trial[ptyp] OR Randomized Controlled Trial[ptyp] OR Research Support, N I H, Extramural[ptyp] OR Research Support, N I H, Intramural[ptyp] OR Research Support, Non U S Gov’t[ptyp] OR Research Support, U S Gov’t, Non P H S[ptyp] OR Research Support, U S Gov’t, P H S[ptyp] OR Research Support, U.S. Government[ptyp]) AND has abstract[text] AND (“humans”[MeSH Terms] OR “animals”[MeSH Terms:noexp])).

**Table 2:** Predefined Clinical Outcomes for Data Extraction

Renal-Related Outcomes	Biochemical-Related Outcomes
Chronic kidney disease	Calcium
eGFR levels	Serum levels
Nephrocalcinosis	Urine levels
Nephrolithiasis/kidney stones	Hypocalcemia
Other terms	Hypercalcemia
Acute kidney injury	Hypercalciuria
Acute renal failure	Phosphate
Acute renal injury	Serum levels
Dehydration	Urine levels
Polyuria	Hyperphosphatemia
Transient renal impairment	Calcium-phosphate product

eGFR, estimated glomerular filtration rate

Duplicate publication abstracts in the search output were removed to create a combined pool of identified articles that were used for inclusion screening.

### Screening and data extraction process

Articles with abstracts were reviewed according to the Table 1 eligibility criteria by two independent reviewers; a third reviewer resolved any nonconsensus selections. Articles excluded were assigned a reason for rejection. Eligible articles that remained after abstract screening underwent a full article review by each of the two independent reviewers to extract all data reported for the 19 predefined relevant clinical outcomes (Table 2). Extracted data were reviewed, and a subset of articles containing data for the most relevant renal-related outcomes (ie, nephrolithiasis/kidney stones, nephrocalcinosis, and CKD) and eGFR levels was selected. One reviewer conducted a second round of extraction, not specified in the protocol, to capture data reported for associations between renal outcomes and predefined biochemical-related outcomes. One reviewer conducted a third round of extraction, not specified in the protocol, to capture available data for thiazide use, blood pressure in the context of reported hypertension, and diabetes mellitus. The individual eligible articles reported data that were collected using differing heterogeneous methodologies that precluded any aggregation of the extracted data and a meta-analysis.

### Articles selected

**Fig. 1:**
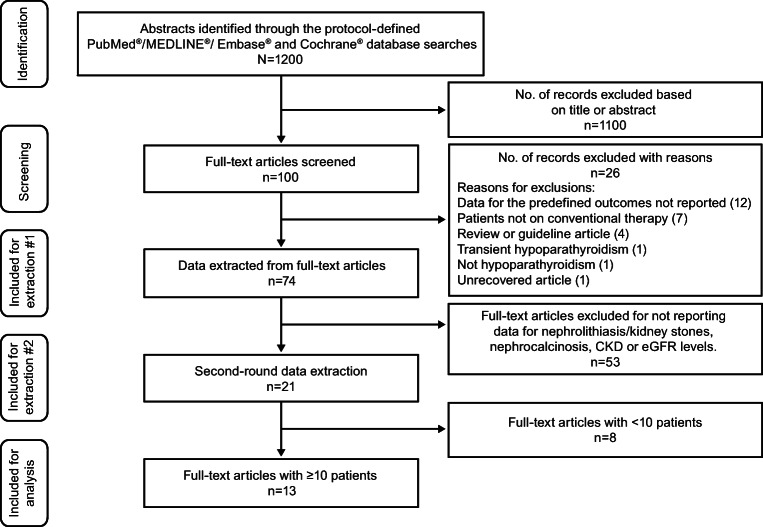
**Flow Diagram of Article Selection for Data Extraction**

The process of the peer-review database search for data of interest yielded 1200 articles (Fig. 1). Following screening and assessment for eligibility, 74 of the 1200 articles had data that reported one or more of the 19 predefined clinical outcomes listed in Table 2. Of the 74 articles with data for one or more of the 19 predefined clinical outcomes, 21 reported data for nephrolithiasis/kidney stones, nephrocalcinosis, CKD, or eGFR levels. Of these 21 papers, 13 articles were the ultimate focus of this review because they reported data for nephrolithiasis/kidney stones, nephrocalcinosis, CKD, or eGFR levels from studies of ≥10 adult (*n* = 11) or pediatric patients (*n* = 2) with chronic hypoparathyroidism.

## Renal calcifications

Treatment of hypoparathyroidism with conventional therapies may result in hypercalciuria, which is a risk factor for nephrolithiasis and nephrocalcinosis [9]. Nephrolithiasis is defined by the appearance of solid calcium-containing stones in the collecting system of the kidney [12–14]. Nephrocalcinosis refers to the parenchymal deposition of calcium salt crystals within the interstitium of the kidney and usually involves the renal medulla [12, 15–17]. Nephrolithiasis and nephrocalcinosis are conditions that may coexist [12, 16]. Nephrolithiasis is commonly diagnosed by abdominal computed tomography (CT) or ultrasonography [18]. Nephrocalcinosis is typically detected using ultrasound imaging as increased bilateral, symmetrical echogenicity within renal pyramids, or by abdominal CT [12, 16].

### Nephrolithiasis/kidney stones

**Fig. 2:**
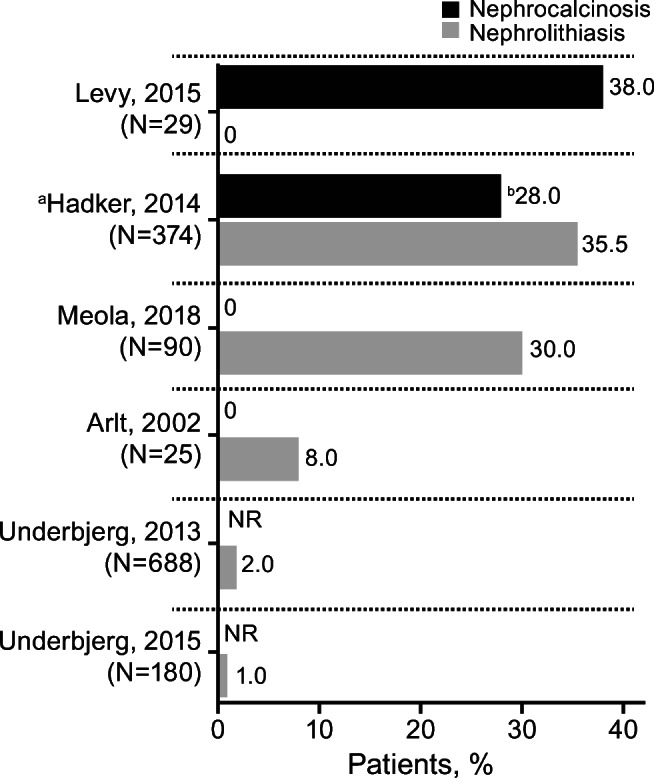
**Percentages of Patients With Renal Calcifications.** Bars and values represent the percentage of patients with nephrolithiasis or nephrocalcinosis. Patient numbers (N) refer to the number of patients with hypoparathyroidism in the study. ^a^Patients self-reporting in a cross-sectional survey. ^b^Included patients with severe hypoparathyroidism (22%) and patients with milder hypoparathyroidism (6%). NR, not reported

Among the six articles with data on the percentage of patients with nephrolithiasis/kidney stones, rates varied from 0% [10] to 35.5% [19] (Table 3**,** Fig. 2**)** [6, 7, 10, 19–21]. Differences in study designs, patient populations, and overall size of the studies may help explain some of the variation in the reported rates of this renal complication. Furthermore, the considerably different assessment methods used in the six studies, ranging from diagnostic codes [6, 7], ultrasound [10, 20, 21], and patient self-reporting [19], likely contributed substantially to the heterogeneity in the reported rates. In the pediatric study of Levy et al., the lowest outcome rate of nephrolithiasis was reported (0%) based on ultrasound [10]. In adult studies, rates were reported using diagnostic codes (1%–2%) [6, 7], ultrasound (8%–30%) [20, 21], and patient self-reporting (35.5%) [19]. These findings suggest that current understanding regarding the frequency of this complication in patients with hypoparathyroidism is likely affected by the choice of diagnostic coding or ultrasound. Meola et al. evaluated a cross-section of patients with chronic hypoparathyroidism on conventional treatment to determine the proportion who met biochemical parameter targets defined by the European Society of Endocrinology (ESE) treatment guidelines [22] in order to meet the ESE treatment goal to relieve symptoms of hypocalcemia and maintain calcium levels in the low or slightly below normal reference range. As part of that study, Meola et al. reported that 30% of the study population had nephrolithiasis detected by renal ultrasound and that most were asymptomatic [21]. Meola et al. used a study design with age- and gender-matched healthy normative controls and determined that the rate and risk of nephrolithiasis/kidney stones was significantly higher in patients with postsurgical chronic hypoparathyroidism versus controls (30% vs 5%, *P* < 0.0001; odds ratio, 8.2). In the two studies that used age- and gender-matched controls, there was an increased risk of nephrolithiasis/kidney stones in patients with postsurgical chronic hypoparathyroidism, but not in patients with nonsurgical chronic hypoparathyroidism (hazard ratios, 4.82 and 0.80, respectively; Table 3**)** [6, 7]. Underbjerg et al. suggest that for renal outcomes there may be an interaction between the age of the individual and duration of disease, with increased risk in older patients [7].

**Table 3:** Nephrolithiasis/Kidney Stones (6 studies)

Article Study Design	Population	Disease Duration/Follow-Up (years)	Supplementation (%)	Methods	Kidney Stones (% of patients)	Reported Association Data Between Those Renal Outcomes and the Predefined Biochemical-Related Outcomes	Serum Calcium	Urinary Calcium	Serum Phosphate	Urine Phosphate	Calcium-Phosphate Product
Underbjerg et al. 2015 [7] Retrospective follow-up study using national health registry data	180 Danish pts with nonsurgical HypoPT, mean age, 49.7 years 540 age- and gender-matched controls	Not reported	Calcium, 71% Active vitamin D analogs, 70%	ICD-8 and ICD-10 codes	1%	Not reported Relevant finding stated in the article: Risk of nephrolithiasis was not increased in pts compared with controls (HR: 0.80 [95% CI, 0.17–3.85])	Not reported	Not reported	Not reported	Not reported	Not reported
							Outcome Hypocalcemia: 27% pts (9 pts)				
Underbjerg et al. 2013 [6] Retrospective follow-up study using national health registry data	688 Danish pts with postsurgical HypoPT, median (range) age, 49 (17–87) years 2064 age- and gender-matched controls	Median (IQR) duration of disease: 8 (4;12)	Calcium, 93% Alfacalcidol, 93%	Determined by ICD-8 or ICD-10 codes	2%	Not reported Relevant finding stated in the article: Compared with controls, pts had increased risk of renal stones HR (unadjusted): 4.82 (95% CI, 2.0–11.64) HR (adjusted for prior renal diseases): 4.22 (1.73–10.30) HR (adjusted for prior diabetes mellitus and renal disease): 4.02 (1.64–9.90)	Not reported	Not reported	Not reported	Not reported	Not reported
Arlt et al. 2002 [20] Cross-sectional study	25 women with postsurgical HypoPT, mean (SD) age, 48.4 (13.6) years 25 sex-, age-, and surgery-matched controls,^a^ mean (SD) age, 49.5 (13.2) years	Median (range) duration of disease: 3 (0.5–38)	Calcium and oral vitamin D, vitamin D metabolites or analogs, 100%	Renal ultrasound	8%	Not reported	2.15 ± 0.21 mmol/L	5.51 ± 4.17 mmol/24 h	1.32 ± 0.22 mmol/L	26.1 ± 8.8 mmol/24 h	Not reported
							Outcome Hypocalcemia^b^: 12% pts (3 pts)	Outcome Hypercalciuria^c^: 23% pts (5/22 pts)			
Meola et al. 2018 [21] Prospective study	90 pts with postsurgical HypoPT Mean (SD) age, females: 50 (14) years; males: 57 (14) years 142 sex- and age-matched healthy normative controls Mean (SD) age, females: 53 (8) years; males: 50 (6) years	Mean ± SD disease duration: 9 ± 7	Calcium, 38.9% Calcitriol, 100%	Renal ultrasound	30%	No significant correlation (*P* = 0.98) between presence of kidney stones and duration of HypoPT, 24-h urinary calcium excretion, total Alb-sCa or vitamin D status	Alb-sCa 8.9 ± 0.5 mg/dL (range 7.5–10.1)	Male: 359 ± 178 mg/24 h Female: 290 ± 155 mg/24 h	3.6 ± 0.7 mg/dL (range 2.2–5.9)	Not reported	Normal, <55 mg^2^/dL^2^ in all pts
							Outcome Hypocalcemia^d^: 14% pts (13 pts)	Outcome Hypercalciuria^e^: Female: 52% pts (33/63 pts) Male: 63% pts (12/19 pts)	Outcome Hyperphosphatemia: 8% pts (7 pts)		
							Hypercalcemia^d^: 20% pts (18 pts)				
Hadker et al. 2014 [19] Patient self-reporting in a cross-sectional survey	374 pts with chronic HypoPT, mean (SD) age, 49.4 (11.6) years	Mean ± SD duration of disease: 12.6 ± 12.4	Calcium, 25% Calcitriol, 44% Ergocalciferol vitamin D_2_ or cholecalciferol vitamin D_3,_ 20% Combination of calcium/calcitriol, 67%	Self-report	35.5% (since diagnosis)	Not reported	Not reported	Not reported	Not reported	Not reported	Not reported
Levy et al. 2015 [10] Long-term retrospective follow-up study	29 *pediatric* pts with chronic HypoPT, mean (SD) age, 11.1 (5.9) years	Mean ± SD duration of disease: 9.1 ± 5.5 Mean ± SD duration of follow-up: 7.4 ± 5.0	Calcitriol/calcium, 100% Cholecalciferol, 79%	Renal ultrasound	0	Not reported	Total calcium: 8.9 ± 0.8 mg/dL Ionized calcium: 4.6 ± 0.5 mg/dL	Average urine calcium/creatinine ratio: 0.27 ± 0.25 mg/mg	5.9 ± 1.2 mg/dL	Not reported	Not reported

Alb-sCa, albumin-corrected serum calcium; ESE, European Society of Endocrinology; HR, hazard ratio; HypoPT, hypoparathyroidism; ICD, international classification of diseases and related health problems; IQR, interquartile range; pt, patient; ULN, upper limit of normal

Note: The following superscripted-letter footnotes are based on information contained in the indicated manuscript

^a^Subtotal thyroidectomy for goiter with intact parathyroid function (*n* = 23) or parathyroid surgery for hyperparathyroidism (*n* = 2)

^b^Below 2.00 mmol/L

^c^>ULN 3–8 mmol/day

^d^ESE target ranges used with hypocalcemia being below the recommended ranges and hypercalcemia above

^e^Values above the ULN (≥300 mg/24 h in males and ≥ 250 mg/24 h in females)

A separate study by Bohrer et al. used a new Likert-scale questionnaire to capture patient self-reported impairment reported as distress level scores [23]. Patients with higher total symptom or distress level scores had more illness manifestations, including kidney stones (plus bone changes, basal ganglia calcification, and cataracts), and had lower serum calcium levels than patients with lower total symptom or distress level scores (both *P* < 0.05).

### Nephrocalcinosis

Among the four articles reporting specifically the percentage of patients with nephrocalcinosis, rates varied from 0% to 38% (Table 4**,** Fig. 2) [10, 19–21]. Similar to the nephrolithiasis/kidney stones outcome, between-study differences in design, methodology and population size may explain the large discrepancies in the reported rates of nephrocalcinosis. In order of lowest to highest, the reported occurrences in the three adult studies used ultrasound (0%) and patient self-reporting (28%); none of the studies reported diagnostic codes [19–21]. Hadker et al. used a patient self-reporting cross-sectional survey of adult patients and found that those who indicated severe disease on the questionnaire (no definition or description of severity grades was provided) reported a significantly higher occurrence of nephrocalcinosis versus patients who reported having mild disease (22% vs 6%; *P* ≤ 0.05) [19].

**Table 4:** Nephrocalcinosis (4 studies)

Article Study Design	Population	Disease Duration/Follow-Up (years)	Supplementation (%)	Methods	Nephrocalcinosis (% of patients)	Reported Association Data Between Those Renal Outcomes and the Predefined Biochemical-Related Outcomes	Serum Calcium	Urinary Calcium	Serum Phosphate	Urine Phosphate	Calcium-Phosphate Product
Arlt et al. 2002 [20] Cross-sectional study	25 women with postsurgical HypoPT, mean (SD) age, 48.4 (13.6) years 25 sex-, age-, and surgery-matched controls,^a^ mean (SD) age, 49.5 (13.2) years	Median (range) duration of disease: 3 (0.5–38)	Calcium and oral vitamin D, vitamin D metabolites or analogs, 100%	Renal ultrasound	0	Not reported	2.15 ± 0.21 mmol/L	5.51 ± 4.17 mmol/24 h	1.32 ± 0.22 mmol/L	26.1 ± 8.8 mmol/24 h	Not reported
							Outcome Hypocalcemia^b^: 12% pts (3 pts)	Outcome Hypercalciuria^c^: 23% pts (5/22 pts)			
Meola et al. 2018 [21] Prospective study	90 pts with postsurgical HypoPT Mean (SD) age, females: 50 (14) years; males: 57 (14) years 142 sex- and age-matched healthy normative controls, mean (SD) age, females: 53 (8) years; males: 50 (6) years	Mean ± SD disease duration: 9 ± 7	Calcium, 38.9% Calcitriol, 100%	Renal ultrasound	0	Not reported	Alb-sCa 8.9 ± 0.5 mg/dL (range 7.5–10.1)	Male: 359 ± 178 mg/24 h Female: 290 ± 155 mg/24 h	3.6 ± 0.7 mg/dL (range 2.2–5.9)	Not reported	Normal, <55 mg^2^/dL^2^ in all pts
							Outcome Hypocalcemia^d^: 14% pts (13 pts)	Outcome Hypercalciuria^e^: Female: 52% pts (33/63 pts) Male: 63% pts (12/19 pts)	Outcome Hyperphosphatemia: 8% pts (7 pts)		
							Hypercalcemia^d^: 20% pts (18 pts)				
Hadker et al. 2014 [19] Patient self reporting in a cross-sectional survey	374 pts with chronic HypoPT, mean (SD) age, 49.4 (11.6) years	Mean ± SD duration of disease: 12.6 ± 12.4	Calcium, 25% Calcitriol, 44% Ergocalciferol vitamin D_2_ or cholecalciferol vitamin D_3,_ 20% Combination of calcium/calcitriol, 67%	Self-report	Pts with severe HypoPT: 22% vs pts with milder HypoPT: 6% (*P* ≤ 0.05)	Not reported	Not reported	Not reported	Not reported	Not reported	Not reported
Levy et al. 2015 [10] Long-term retrospective follow-up study	29 *pediatric* pts with chronic HypoPT, mean (SD) age, 11.1 (5.9) years	Mean ± SD duration of disease: 9.1 ± 5.5 Mean ± SD duration of follow-up: 7.4 ± 5.0	Calcitriol/calcium, 100% Cholecalciferol, 79%	Renal ultrasound	38%	Multivariate analysis: degrees of relative hypercalcemia^f^ and hyperphosphatemia^g^ most significant predictors for nephrocalcinosis (R^2^ = 0.47, *P* < 0.01) Relevant finding stated in the article: Nephrocalcinosis resolved after initial ultrasound (*n* = 2); remained in early stage I (*n* = 3), progressed to stage III (*n* = 6) Pts with non-resolved (*n* = 9) vs w/o (*n* = 18) nephrocalcinosis had a greater: degree of hypercalcemia^f^ (*P* = 0.005); degree of hypocalcemia^h^ (*P* = 0.004); duration of hypocalcemia (*P* = 0.003); degree of hyperphosphatemia^g^ (*P* = 0.01)	Total calcium: 8.9 ± 0.8 mg/dL Ionized calcium: 4.6 ± 0.5 mg/dL Total calcium: Pts with nephrocalcinosis: 8.5 ± 1.1 mg/dL Pts w/o nephrocalcinosis: 9.2 ± 0.6 mg/dL	Average urine calcium/creatinine ratio: 0.27 ± 0.25 mg/mg	5.9 ± 1.2 mg/dL Pts with nephrocalcinosis: 6.0 ± 1.9 mg/dL Pts w/o nephrocalcinosis: 5.8 ± 0.9 mg/dL	Not reported	Not reported
							Outcome Hypocalcemia^i^: Percentage of time with total calcium <8.0 mg/dL: Pts with nephrocalcinosis: 29.4 ± 20.4% Pts w/o nephrocalcinosis: 10.5 ± 11.3%		Outcome Hyperphosphatemia^k^: Percentage of time with phosphate concentrations above age-adjusted levels: Pts with nephrocalcinosis: 50 ± 36.2% Pts w/o nephrocalcinosis: 29 ± 29.4%		
							Outcome Hypercalcemia^j^: Percentage of time with total calcium >9.6 mg/dL: Pts with nephrocalcinosis: 22.8 ± 23.8% Pts w/o nephrocalcinosis: 35.3 ± 31.7%				

Alb-sCa, albumin-corrected serum calcium; AUC, area under the curve; HypoPT, hypoparathyroidism; pt, patient; ULN, upper limit of normal

Note: the following superscripted-letter footnotes are based on information contained in the indicated manuscript

^a^Subtotal thyroidectomy for goiter with intact parathyroid function (*n* = 23) or parathyroid surgery for hyperparathyroidism (*n* = 2)

^b^Below 2.00 mmol/L

^c^>ULN 3–8 mmol/day

^d^ESE target ranges used with hypocalcemia being below the recommended ranges and hypercalcemia above

^e^Values above the ULN (≥300 mg/24 h in males and ≥ 250 mg/24 h in females)

^f^AUC of total calcium concentrations >9.6 mg/dL

^g^AUC above age-adjusted phosphate levels

^h^AUC of total calcium concentrations <8.0 mg/dL

^i^Percentage of time with total calcium <8.0 mg/dL

^j^Percentage of time with total calcium >9.6 mg/dL

^k^Percentage of time with phosphate concentrations above age-adjusted levels

Levy et al. pediatric study that used renal ultrasound reported 38% of patients had nephrocalcinosis, in contrast to the finding that no patients had nephrolithiasis/kidney stones [10]. This study also enabled evaluation of changes in nephrocalcinosis using a staging system developed by Boyce et al., in which stage 0 was no echogenicity; stage I, mild echogenicity around medullary pyramid borders; stage II, moderate echogenicity around and inside pyramids; and stage III, severe echogenicity of entire pyramids [12]. Of the 11 patients with nephrocalcinosis after the initial ultrasound, the nephrocalcinosis resolved in two patients (18%), remained in early stage I in three patients (27%), and progressed from stage I to III in six patients (55%). In the two patients with resolved nephrocalcinosis, both had DiGeorge syndrome, and calcium concentrations were more frequently within the target range versus patients in whom nephrocalcinosis persisted (81% ± 7.6% vs 56% ± 8.5%; *P* = 0.01).

### Combined data for nephrolithiasis and/or nephrocalcinosis

Four articles reported data on the percentage of patients with nephrolithiasis and/or nephrocalcinosis as a combined outcome; all studies used ultrasound or CT scans [9, 24–26]. Among the four studies, the rates were similar to those reported in the studies using separated nephrolithiasis and nephrocalcinosis outcomes (19% to 31%; Table 5). In a study by Leidig-Bruckner et al. of 33 patients with postsurgical hypoparathyroidism (and medullary thyroid carcinoma), there were two cases noted for hospitalization for symptomatic nephrolithiasis [26]. The article also provided details about the nine patients (27%) with documented renal calcifications; five patients had initially received high cholecalciferol dosages, and two patients had received dihydrotachysterol. Also, three of the nine patients receiving cholecalciferol/dihydrotachysterol experienced transient renal failure.

**Table 5:** Nephrolithiasis and/or Nephrocalcinosis (4 studies)

Article Study Design	Population	Disease Duration/Follow-Up (years)	Supplementation	Methods	Nephrolithiasis and/or Nephrocalcinosis (% of patients)	Reported Association Data Between Those Renal Outcomes and the Predefined Biochemical-Related Outcomes	Serum Calcium	Urinary Calcium	Serum Phosphate	Urine Phosphate	Calcium-Phosphate Product
Lopes et al. 2016 [25] Retrospective observational study	55 pts with chronic HypoPT, mean (SD) age, 44.5 (19.3) years 41 (74.5%) with post-surgical HypoPT 5 (9.1%) with pseudoHypoPT 9 (16.4%) with autoimmune HypoPT	Mean ± SD duration of disease: 11.2 ± 7.5 (range 1–32)	Calcium, 92% Calcitriol, 80% Cholecalciferol, 75%	Renal ultrasound	25% (10/40 with imaging)	No correlation between serum and urinary levels of calcium and the presence of calcification Relevant finding stated in the article: Weight-adjusted urinary calcium in 24 h was higher in pts with renal calcification vs those without (3.3 mg/kg/d vs 1.8, respectively; *P* < 0.05)	6.87–8.62 mg/dL (mean, first to last visit)	Outcome Hypercalciuria^a^: 27% pts (15 pts)	6.14–4.89 mg/dL(mean, first to last visit)	Not reported	Not reported
Leidig-Bruckner et al. 2016 [26] Retrospective, longitudinal chart review	33 pts with medullary thyroid carcinoma and postsurgical HypoPT, mean (SD) age 52.8 (13.7) years: Classified as having partial HypoPT^b^ (*n* = 20) or complete HypoPT^b^ (*n* = 13)	Mean ± SD duration of disease: 15.9 ± 9.4 Mean ± SD follow-up: 11.9 ± 6.6	Calcium, 72.7% Cholecalciferol, 18.1% Calcitriol, 33.3% Alfacalcidol, 6.1% Dihydrotachysterol, 18.2%	Radiological imaging (ultrasound, CT, and/or MRI) Calcification group: documented calcifications, renal stones, medullary sponge kidney	27% Partial HypoPT^b^: 25% Complete HypoPT^b^: 31% 2 pts hospitalized for symptomatic nephrolithiasis	Not reported Relevant finding stated in the article: Incidence was higher in pts who initially received high cholecalciferol dosages Of the 9 pts with renal calcifications, 2 were treated with calcitriol from the beginning of treatment, 5 initially received high cholecalciferol doses, and 2 received dihydrotachysterol [see Table 6 for the reported eGFR data and renal calcifications]	Partial HypoPT^b^: 2.13 ± 0.10 mmol/L Complete HypoPT^b^: 2.12 ± 0.12 mmol/L	Partial HypoPT^b^: 3.13 ± 1.9 mmol/L (range 1–10, *n* = 17, end of study) Complete HypoPT^b^: 5.20 ± 3.22 mmol/L (range 1–10, *n* = 10, end of study)	Partial HypoPT^b^: 1.4 ± 0.18 mmol/L Complete HypoPT^b^: 1.51 ± 0.22 mmol/L	Not reported	Partial HypoPT^b^: 2.98 ± 0.32 mmol^2^/L^2^ Complete HypoPT^b^: 3.16 ± 0.42 mmol^2^/L^2^
							Outcome Hypocalcemia: 27% pts (9 pts)				
Mitchell et al. 2012 [9] Retrospective, longitudinal chart review	120 pts with chronic HypoPT mean (SD) [range] age, 52 (19) [2–87] years	Mean ± SD duration of disease: 17 ± 16 (range 1–59) Mean ± SD follow-up: 7.4 ± 5.1	Calcium, 94% Calcitriol, 88% High-dose vitamin D, 6% Thiazide, 20% Relevant finding stated in the article: Pts on a thiazide diuretic had higher urinary calcium levels (mean 318 vs 197 mg, *P* = 0.02)	Renal/abdominal ultrasound and abdominal CT	31% (17/54 with imaging)	2 pts required renal transplant because of nephrocalcinosis	Mean±SD 8.6 ± 1.1 mg/dL (range 5.3–11.5)	Mean±SD 216 ± 140 mg/24 h (range 8–557)	Mean±SD 4.2 ± 0.9 (range 1.3–7.8) mg/dL	Not reported	Mean±SD 35.4 ± 9.0 mg^2^/dL^2^ 22% pts: >55 mg^2^/dL^2^ (at least once during study period)
							Outcome Hypocalcemia: 16% pts (most recent measurement)	Outcome Hypercalciuria: 38% pts overall 26% pts (most recent measurement)			
							Outcome Hypercalcemia: 13% pts (most recent measurement) Frank hypercalcemia^c^: 2% pts 3 episodes of mild hypercalcemia associated with elevated 25-OH vitamin D levels				
Kim et al. 2015 [24] Retrospective	37 *pediatric* pts with primary HypoPT median (range) age, 1.7 months (1 day–17 years)	Mean ± SD duration of follow-up: 7.0 ± 5.3 (range 0.5–22)	Calcium and calcitriol or calcitriol alone, 57%	Renal ultrasound in 26 pts (conducted every ~2.5 years)	19%	Not reported Relevant finding stated in the article: Developed after 3.5 years (range 1.6–12.5) after calcium and calcitriol supplementation	Total Ca: 2.1 ± 0.2 mmol/L (range 1.8–2.5) Ionized Ca: 1.1 ± 0.1 mmol/L(range 0.9–1.4)	Not reported	1.7 ± 0.3 mmol/L (range 1.3–2.2)	Not reported	Not reported

CT, computed tomography; eGFR, estimated glomerular filtration rate; HypoPT, hypoparathyroidism; MRI, magnetic resonance imaging; pt, patient; PTH, parathyroid hormone

Note: the following superscripted-letter footnotes are based on information contained in the indicated manuscript

^a^>250 mg/24 h for females and > 300 mg/24 h for males

^b^Partial hypoparathyroidism defined ≥1 PTH measurement >10 ng/L; complete hypoparathyroidism defined as all PTH measurements ≤10 ng/L

^c^>10.5 mg/dL

## Chronic kidney disease

**Fig. 3:**
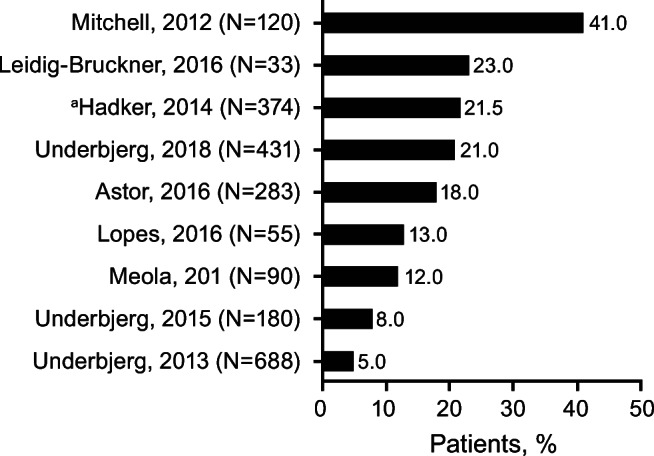
**Percentages of Patients With Chronic Kidney Disease.** Bars and values represent the percentage of patients with chronic kidney disease determined by eGFR <60 mL/min/1.73 m^2^, ≥ stage 3, or renal insufficiency ICD-8 and ICD-10 codes. The methods used by each study are detailed in Table 6. Patient numbers (N) refer to the number of patients with hypoparathyroidism in the study. ^a^Patients self-reporting in a cross-sectional survey. eGFR, estimated glomerular filtration rate; ICD, international classification of diseases and related health problems

Data for CKD, renal insufficiency, and eGFR levels were reported in 10 articles [6–10, 19, 21, 25–27]. Eight adult studies reported the percentage of patients with CKD based on standard methods of eGFR <60 mL/min/1.73 m^2^ or  ≥ stage 3 classification [28], or renal insufficiency international classification of diseases (ICD) ICD-8 and ICD-10 codes. One survey reported CKD based on adult patients self-reporting for chronic kidney failure [19]. The pediatric study of Levy et al. used the revised Schwartz estimating equation for nonchronic kidney disease populations. The methods used by each study are detailed in Table 6. The rates of CKD varied among studies from 2.5% to 41% (Fig. 3 and Table 6).

**Table 6:** Chronic Kidney Disease and eGFR Levels (10 studies)

Article Study Design	Population	Disease Duration/Follow-Up (years)	Supplementation	Methods	CKD (% of Patients)	Reported Association Data Between Those Renal Outcomes and the Predefined Biochemical-Related Outcomes	Serum Calcium	Urinary Calcium	Serum Phosphate	Urine Phosphate	Calcium-Phosphate Product
Hadker et al. 2014 [19] Patient self-reporting in a cross-sectional survey	374 pts with chronic HypoPT, mean (SD) age, 49.4 (11.6) years	Mean ± SD duration of disease: 12.6 ± 12.4	Calcium, 25% Calcitriol, 44% Ergocalciferol vitamin D_2_ or cholecalciferol vitamin D_3,_ 20% Combination of calcium/calcitriol, 67%	Self-report; CKD reported as chronic kidney failure	CKD 2.5% with mild HypoPT^a^ vs 19% with severe HypoPT^a^ (*P* ≤ 0.05)	Not reported	Not reported	Not reported	Not reported	Not reported	Not reported
Underbjerg et al. 2013 [6] Retrospective follow-up study using national health registry data	688 Danish pts with postsurgical HypoPT, median (range) age, 49 (17–87) years 2064 age- and gender-matched controls	Median (IQR) duration of disease: 8 (4;12)	Calcium, 93% Alfacalcidol, 93%	Reported as renal insufficiency defined using ICD codes	5% (35 pts)	Not reported Relevant finding stated in the article: Compared with controls, pts had increased risk of renal insufficiency HR (unadjusted): 4.95 (95% CI, 2.88–8.50) HR (adjusted for prior renal diseases): 4.54 (2.63–7.84) HR (adjusted for prior diabetes mellitus and renal disease): 3.10 (1.73–5.55)	Not reported	Not reported	Not reported	Not reported	Not reported
Underbjerg et al. 2015 [7] Retrospective follow-up study using national health registry data	180 Danish pts with nonsurgical HypoPT, mean age, 49.7 years 540 age- and gender-matched controls	Not reported	Calcium, 71% Active vitamin D analogs, 70%	Reported as renal insufficiency, defined using ICD codes	8% (15 pts)	Not reported Relevant finding stated in the article: Compared with controls, pts had increased risk of renal insufficiency HR (unadjusted): 6.01 (95% CI, 2.45–14.75)	Not reported	Not reported	Not reported	Not reported	Not reported
							Outcome Hypocalcemia: 27% pts (9 pts)				
Meola et al. 2018 [21] Prospective study	90 pts with postsurgical HypoPT Mean (SD) age, females: 50 (14) years; males: 57 (14) years 142 sex- and age-matched healthy normative controls, mean (SD) age, females: 53 (8) years; males: 50 (6) years	Mean ± SD disease duration: 9 ± 7	Calcium, 38.9% Calcitriol, 100%	CKD-EPI equation	<60 mL/min/1.73 m^2^ 12% pts (11 pts) Mean ± SD 82 ± 20 mL/min/1.73 m^2^ (range 33–148)	Not reported	Alb-sCa 8.9 ± 0.5 mg/dL (range 7.5–10.1)	Male: 359 ± 178 mg/24 h Female: 290 ± 155 mg/24 h	3.6 ± 0.7 mg/dL (range 2.2–5.9)	Not reported	Normal, <55 mg^2^/dL^2^ in all pts
							Outcome Hypocalcemia^b^: 14% pts (13 pts)	Outcome Hypercalciuria^c^: Females: 52% pts (33/63 pts) Males: 63% pts (12/19 pts)	Outcome Hyperphosphatemia: 8% pts (7 pts)		
							Outcome Hypercalcemia^b^: 20% pts (18 pts)				
Astor et al. 2016 [27] Pt survey using hospital registry	283 pts with chronic HypoPT in Norway, median (range) age, 53 (9–89) years 25% pts (70/283 pts) Nonsurgical HypoPT 70% pts (197/283 pts) Postsurgical HypoPT 6% pts (16/283 pts) PseudoHypoPT	Not reported	Calcium, 70% Calcitriol, 40% Alphacalcidiol, 44% Ergocalciferol, 19% Cholecalciferol, 29%	MDRD formula: calculated eGFR (MDRD formula) × (0.20247 × height (m)^0.725^ × weight (kg)^0.425^ )/1.73, where the MDRD formula is 175 × (s-Creatinine/88.4) ^−1.154^ × (age)^−0.203^ × 0.742 (if female)	<60 mL/min/1.73 m^2^ 18% pts (51 pts) Median eGFR 80.8 mL/min/1.73 m^2^ (range 14.6–215.7)	Not reported Relevant findings stated in the article: Despite conventional therapy, 18% had kidney failure (eGFR <60 mL/min/1.73 m^2^), of whom 98% had an eGFR level > 30 mL/min/1.73 m^2^	Alb-sCa 2.08 mmol/L (range 1.47–2.84)	0.51 mmol/mmol creatinine (range 0.02–2.29)	1.29 mmol/L (range 0.76–2.55)	Not reported	Not reported
Underbjerg et al. 2018 [8] Case-controlled retrospective study using national health registry data	431 Danish pts with postsurgical or nonsurgical HypoPT, mean (range) age, 41 (0–87) years	Median (range) duration of disease: 12.7 (0.5–87.1)	Calcium, 95.3% Alfacalcidol, 94.4%	MDRD equation [sex-specific eGFR using MDRD equation, converted to stages of CKD according to criteria defined by the NKF] eGFR <60 mL/min/1.73 m^2^ as threshold limit for renal insufficiency	<60 mL/min/1.73 m^2^ 21% pts (91 pts) 60–90 mL/min/1.73 m^2^ 45% pts (194 pts) >90 mL/min/1.73 m^2^ 34% pts (147 pts)	Not reported	Time-weighted avg^d^: Ionized Ca 1.17 mmol/L (range 1.14–1.21 (431 pts)	Not reported	Time-weighted avg^d^ 1.21 mmol/L(range 1.11–1.32) (353 pts)	Not reported	Time-weighted avg^d^ 2.80 mmol^2^/L^2^ (range 2.51–3.03) (304 pts)
							Outcome Hypercalcemia: ≥1 episodes 41% pts (177/431 pts); ≥ 4 episodes 13% pts (58/431 pts)		Outcome Hyperphosphatemia: 7% pts (26 pts)		
Leidig-Bruckner et al. 2016 [26] Retrospective, longitudinal chart analysis	33 with medullary thyroid carcinoma and postsurgical HypoPT, mean (SD) age, 52.8 (13.7) years: classified as having partial HypoPT^e^ (*n* = 20) or complete HypoPT^e^ (*n* = 13)	Mean ± SD duration of disease: 15.9 ± 9.4 Mean ± SD follow-up: 11.9 ± 6.6	Calcium, 72.7% Cholecalciferol, 18.1% Calcitriol, 33.3% Alfacalcidol, 6.1% Dihydrotachysterol, 18.2%	Cockcroft-Gault formula	<60mL/min/ 1.73 m^2^ Partial HypoPT^e^: 5% pts (1 pt) Complete HypoPT^e^: 23% pts (3 pts) >90 mL/min/1.73 m^2^ Partial HypoPT^e^: 45% pts (9 pts) Complete HypoPT^e^: 61.5% (8 pts)	Not reported Relevant findings stated in the article: The eGFR was negatively correlated with the duration of hypoparathyroidism (r = −0.62; *P* = 0.0001). This correlation remained significant after adjusting for chronological age (partial correlation, adjusted for age r = −0.35, *P* = 0.04). The correlation between eGFR and duration of hypoparathyroidism was independent from the degree of hypoparathyroidism (partial or complete) and also independent from the radiological presence of calcification More pts with calcifications had eGFR <60 mL/min/1.73 m^2^ (ie, CKD) 22% (2/9 pts) than those without calcifications 8% (2/24 pts); differences were not significant At last visit, eGFR was lower in pts with calcifications (9/33 pts) than in those without calcifications (24/33 pts) (77 ± 17 vs 95 ± 29 mL/min/1.73 m^2^; *P* = 0.07)	Partial HypoPT^e^: 2.13 ± 0.10 mmol/L Complete HypoPT^e^: 2.12 ± 0.12 mmol/L	Partial HypoPT^e^: 3.13 ± 1.9 mmol/L (*n* = 17) Complete HypoPT^e^: 5.20 ± 3.22 mmol/L (*n* = 10)	Partial HypoPT^e^: 1.4 ± 0.18 mmol/L Complete HypoPT^e^: 1.51 ± 0.22 mmol/L	Not reported	Partial HypoPT^e^: 2.98 ± 0.32 mmol^2^/L^2^ Complete HypoPT^e^: 3.16 ± 0.42 mmol^2^/L^2^
							Outcome Hypocalcemia: 27% pts (9 pts)				
Lopes et al. 2016 [25] Retrospective observational study	55 pts with chronic HypoPT, mean (SD) age, 44.5 (19.3) years 41 (74.5%) with postsurgical HypoPT, 5 (9.1%) with pseudoHypoPT, and 9 (16.4%) with autoimmune HypoPT	Mean ± SD duration of disease: 11.2 ± 7.5 (range 1–32)	Calcium, 92% Calcitriol, 80% Cholecalciferol, 75%	Cockcroft-Gault formula (for patients with weight and creatinine available for the last visit) CKD stages per KDIGO	CKD Stage 2 33% pts (15 pts) Stage 3 9% pts (4 pts) Stage 4 2% pts (1 pt) Stage 5 2% pts (1 pt) Mean ± SD 92.9 ± 36.2 mL/min/1.73 m^2^ (range 14–223)	Not reported	6.87–8.62 mg/dL (mean, first to last visit)	Outcome Hypercalciuria^f^: 27% pts (15 pts)	6.14–4.89 mg/dL (mean, first to last visit)	Not reported	Not reported
Mitchell et al. 2012 [9] Retrospective, longitudinal chart review	120 pts with chronic HypoPT, mean (SD) [range] age, 52 (19) [2–87] years	Mean ± SD duration of disease: 17 ± 16 (range 1–59) Mean ± SD follow-up: 7.4 ± 5.1	Calcium, 94% Calcitriol, 88% High-dose vitamin D, 6% Thiazide, 20% Relevant finding stated in the article: Pts on a thiazide diuretic had higher urinary calcium levels (mean 318 vs 197 mg, *P* = 0.02)	MDRD equation	<60 mL/min/1.73 m^2^ 41% pts (44/107 pts) This parameter analysis had age-matched normative controls	eGFR Univariate analyses: age (*P* < 0.001), duration of disease (*P* < 0.001), avg_tw_ calcium (*P* < 0.001), and estimated proportion of time with serum calcium higher than 9.5 mg/dL (*P* < 0.001) negatively correlated with eGFR Multivariate regression analyses: age (*P* < 0.001), duration of disease (*P* = 0.032), and proportion of time with relative hypercalcemia (*P* = 0.005) remained significantly associated with eGFR	Mean±SD 8.6 ± 1.1 mg/dL (range 5.3–11.5)	Mean± SD 216 ± 140 mg/24 h (range 8–557)	Mean±SD 4.2 ± 0.9 (range 1.3–7.8) mg/dL	Not reported	Mean±SD 35.4 ± 9.0 mg^2^/dL^2^ 22% pts (25 pts): >55 mg^2^/dL^2^ (at least once during study period)
							Outcome Hypocalcemia: 16% pts (most recent measurement)	Outcome Hypercalciuria: 38% pts overall 26% pts (most recent measurement)			
							Outcome Hypercalcemia**:** 13% pts (most recent measurement) Frank hypercalcemia^g^: 2% pts 3 episodes of mild hypercalcemia associated with elevated 25-OH vitamin D levels				
Levy et al. 2015 [10] Long-term retrospective follow-up study	29 *pediatric* pts with chronic HypoPT, mean (SD) age, 11.1 (5.9) years	Mean ± SD duration of disease: 9.1 ± 5.5 Mean ± SD duration of follow-up: 7.4 ± 5.0	Calcitriol/calcium, 100% Cholecalciferol, 79%	eGFRRevised Schwartz estimating equation for nonchronic kidney disease populations	<60 mL/min/1.73 m^2^ 0% pts (0 pts) >60 mL/min/1.73 m^2^ 100% pts (29 pts) 60–90 mL/min/1.73 m^2^ 45% pts (13 pts) Mean ± SD 92 ± 18 mL/min/1.73 m^2^ Males: Mean ± SD 85.1 ± 11.9 mL/min/1.73 m^2^ Females: Mean ± SD 99.3 ± 20.4 mL/min/1.73 m^2^	Univariate analysis: Higher calcium concentrations (r = −0.42, *P* = 0.02) and a greater percentage of time with total calcium >9.6 mg/dL (r = −0.41, *P* = 0.03) were associated with lower eGFR	Total calcium: 8.9 ± 0.8 mg/dL Ionized calcium: 4.6 ± 0.5 mg/dL	Average urine calcium/creatinine ratio: 0.27 ± 0.25 mg/mg	5.9 ± 1.2 mg/dL	Not reported	Not reported

Alb-sCa, albumin-corrected serum calcium; CKD, chronic kidney disease; CKD-EPI, Chronic Kidney Disease Epidemiology Collaboration; eGFR, estimated glomerular filtration rate; ESE, European Society of Endocrinology; HR, hazard ratio; HypoPT, hypoparathyroidism; ICD codes, international statistical classification of diseases and related health problems; IQR, interquartile range; KDIGO, Kidney Disease Outcomes Quality Initiative; MDRD, Modification of Diet in Renal Disease; NKF, National Kidney Foundation; NR, not reported; PTH, parathyroid hormone; ULN, upper limit of normal

Note: the following superscripted-letter footnotes are based on information contained in the indicated manuscript

^a^HypoPT severity was self-reported

^b^ESE target ranges used with hypocalcemia being below the recommended ranges and hypercalcemia above

^c^Values above the ULN (≥300 mg/24 h in males and ≥ 250 mg/24 h in females)

^d^From first available biochemical measurement after index date to end of follow-up

^e^Partial hypoparathyroidism defined ≥1 PTH measurement >10 ng/L; complete hypoparathyroidism defined as all PTH measurements ≤10 ng/L

^f^>250 mg/24 h for females and > 300 mg/24 h for males

^g^>10.5 mg/dL

In a survey by Hadker et al., 2.5% of patients with milder hypoparathyroidism symptoms and 19% of patients with severe hypoparathyroidism symptoms self-reported having CKD [19]. In the two studies that used age- and gender-matched controls, there was an increased risk of renal insufficiency in both patients with postsurgical or nonsurgical chronic hypoparathyroidism (hazard ratios, 4.95 and 6.01, respectively; Table 6) [6, 7].

There were five adult studies that reported eGFR data using either the Chronic Kidney Disease Epidemiology Collaboration (CKD-EPI) equation, Cockcroft-Gault (eCrCl) or Modification of Diet in Renal Disease (MDRD) formula [8, 9, 21, 26, 27]. Four articles reported similar percentages of patients with eGFR <60 mL/min/1.73 m^2^, ranging from 12% to 23% of patients; the overall populations had a mean duration of disease of 9 to 15.9 years [8, 21, 26, 27]. In a study by Mitchell et al., 41% of 120 patients had eGFR <60 mL/min/1.73 m^2^; the overall population had a mean duration of disease of 17 years [9].

## Thiazide use, hypertension, and diabetes mellitus

Renal outcomes may be affected by thiazide use, blood pressure in the context of reported hypertension, and diabetes mellitus. However, an examination of the 13 articles in this review found that very few reported any data on the following indices: thiazide use (*n* = 2) [9, 21], blood pressure in the context of reported hypertension (*n* = 2) [8, 21], and diabetes mellitus (*n* = 3) [6, 8, 21]. In the two studies that reported thiazide data, the percentages of patients prescribed this medication were 2% and 20%; the authors of the paper with the higher percentage noted they were unable to determine whether the patients were prescribed thiazides for hypertension, hypercalciuria, or both [9, 21]. In the two studies that reported the percentage of patients with hypertension or diabetes mellitus type 1 or 2, ranges were 3.5% to 18% and 1% to 8.4%, respectively [8, 21]. The authors of these four articles did not infer any relationship with renal outcomes based on the limited data. These conditions may have been underreported in the articles because they were not the primary focus of the publications and these diagnoses were based on hospital records.

## Associations between renal outcomes and biochemical or disease parameters

The collation of the predefined renal outcomes and disease-relevant biochemical parameters affords the opportunity to reveal relationships not previously described. However, it was recognized that there are limitations to this approach in that an individual study with single biochemical measures (24-h urine collection) may not characterize the longitudinal status of that parameter.

The association of nephrolithiasis/kidney stones with a number of biochemical or clinically relevant parameters was only reported by one study of 90 patients [21]. No significant correlation (*P* = 0.98) was seen between the presence of kidney stones and the duration of hypoparathyroidism, 24-h urinary calcium excretion, total albumin-corrected serum calcium, or vitamin D status (Table 3).

Association data with nephrocalcinosis and a number of biochemical or clinically relevant parameters were reported by the Levy et al. pediatric study (*n* = 29) [10]. In a multivariate analysis, the most significant predictors for nephrocalcinosis were the degree of relative hypercalcemia (area under the curve [AUC] of total calcium concentrations >9.6 mg/dL) and the degree of hyperphosphatemia (AUC above age-adjusted phosphate concentrations; R^2^ = 0.47, *P* < 0.01; Table 4). Odds ratios for the association between nephrocalcinosis and degree of hypercalcemia and degree of hyperphosphatemia were 1.027 (95% CI, 1.003–1.052) and 1.004 (95% CI, 1.001–1.008), respectively. Compared with 18 patients in the study without nephrocalcinosis, the nine patients with unresolved nephrocalcinosis had a greater degree of hypercalcemia (AUC of total serum calcium concentrations >9.6 mg/dL; *P* = 0.005), hyperphosphatemia (AUC above age-adjusted phosphate; *P* = 0.01), and hypocalcemia (AUC of total serum calcium concentrations <8.0 mg/dL; *P* = 0.004) and a greater duration of hypocalcemia (percentage of time with total calcium <8.0 mg/dL; *P* = 0.003).

Association data with the combined nephrolithiasis and/or nephrocalcinosis outcome and biochemical parameters were only reported in a study by Lopes et al. of 55 patients [25]. Weight-adjusted 24-h urinary calcium was higher in patients with renal calcification versus those without (3.3 vs 1.8 mg/kg/day, respectively; *P* < 0.05; Table 5). However, there was no correlation between serum and urinary levels of calcium and the presence of calcification.

Mitchell et al. in their study of 120 patients examined correlations between CKD and a number of biochemical or clinically relevant parameters; eGFR <60 mL/min/1.73 m^2^ was compared with age-matched normative controls (Table 6) [9]. Univariate analyses found that eGFR levels were negatively correlated with age (*P* < 0.001), duration of disease (*P* < 0.001), average time-weighted serum calcium (*P* < 0.001), and estimated proportion of time with serum calcium higher than 9.5 mg/dL (*P* < 0.001). Average time-weighted serum phosphate and average calcium-phosphate product were not correlated with eGFR levels. Multivariate regression analyses of the predictors from the univariate analyses found that eGFR levels remained significantly associated with age (*P* < 0.001), duration of disease (*P* = 0.032), and proportion of time with relative hypercalcemia (*P* = 0.005). No association was seen between eGFR levels and either 24-h urine calcium values or presence of renal calcification. In a univariate analysis of the pediatric study, lower eGFR was associated with higher calcium concentrations (r = −0.42, *P* = 0.02) and a greater proportion of time with relative hypercalcemia (r = −0.41, *P* = 0.03) [10].

In a case-controlled retrospective study of 431 patients with national health registry data of long-term complications, Underbjerg et al. applied a composite endpoint of renal stones (defined by ICD codes) and renal insufficiency (defined by eGFR <60 mL/min/1.73 m^2^) to describe renal disease in patients with hypoparathyroidism [8]. This study showed that a decreased risk of any renal disease was associated with a higher dose of alfacalcidol supplementation (>1 vs ≤1 μg/day; *P =* 0.03). An increased risk of any renal disease was associated with an increased serum calcium-phosphate product (>2.80 mmol^2^/L^2^), increased number of hypercalcemic episodes, and long duration of disease. Predictors of any incidence of renal disease were disease duration (≥12.7 vs <12.7 years; *P* < 0.01) and increased calcium-phosphate product (≤2.80 vs >2.80 mmol^2^/L^2^; *P* < 0.01). Although the articles used divergent outcome measures and methodologies to assess biochemical parameters, they nevertheless revealed valuable insights into factors associated with renal outcomes in patients with chronic hypoparathyroidism.

## Limitations

There was a significant heterogeneity in the data and in the methods of reporting data for each of the renal outcomes within published articles of clinical data studies of adult and pediatric patients with chronic hypoparathyroidism. Given the relatively low prevalence of hypoparathyroidism, it is not surprising that there are large gaps in the reporting of the key disease-related biochemical parameters studies of patients with chronic hypoparathyroidism; prospective studies are needed to address these knowledge gaps. The methodology for the collection of CKD information based on eGFR data was heterogeneous and often unclear. Only one study explicitly reported a collection method according to the CKD definition (ie, low eGFR levels on ≥2 occasions with an interval of ≥3 months) [26]. However, the low eGFR rate reported in the majority of the articles suggests a common comorbidity in patients with chronic hypoparathyroidism. There are limitations intrinsic to the detection method used. For example, it is possible that imaging for the assessment of nephrolithiasis and nephrocalcinosis may have selected patients who were at higher risk of developing these conditions. These limitations precluded a meta-analysis with data extracted from the selected studies.

## Discussion

This systematic review of the literature found evidence that patients with chronic hypoparathyroidism managed with conventional therapy of oral calcium and active vitamin D supplementation have adverse renal outcomes of nephrolithiasis/kidney stones, nephrocalcinosis, and CKD. While there was a wide range for the frequency rate of each outcome, generally one-third of the patients had these renal complications.

Compared with publications on the general population, rates of nephrolithiasis (up to 36%) and CKD (up to 41%) are higher in patients with chronic hypoparathyroidism. Romero et al. reported overall population nephrolithiasis prevalence data from five countries ranging from 2% to 15% [29]. Of note, all studies reporting separate outcomes for nephrolithiasis and nephrocalcinosis used diagnostic codes or kidney ultrasound. The latter method has limited ability to detect kidney stones of a smaller size that may in part depend on the operator; therefore, the true prevalence of kidney stones may be underestimated using the ultrasound technique [30]. The range of rates reported in the two pediatric studies for nephrolithiasis, nephrocalcinosis, or the combined outcome may reflect the difficulty in distinguishing between small stones and parenchymal calcifications. We are unable to provide an obvious explanation for the dramatic difference in the rates of nephrolithiasis and nephrocalcinosis between the Meola et al. adult study and the Levy et al. pediatric study, but we speculate that it was a classification choice by each study group. It is our opinion that this differing classification does not detract from the collective data indicating an increased risk of these renal complications. Epidemiologic data from the Global Burden of Disease study reported a 4% age-standardized prevalence rate for CKD [31]. Only four studies had age- and gender-matched control groups, which is an important limitation to consider when making any cross-study comparisons, or with rates of renal outcomes in the general population [6, 7, 20, 21]. Three of the four studies found an increased risk of nephrolithiasis in patients with postsurgical chronic hypoparathyroidism compared with patients with nonsurgical chronic hypoparathyroidism or general population controls [6, 7, 21]. In two of the four studies, there was an increased risk of renal insufficiency in patients with either postsurgical or nonsurgical chronic hypoparathyroidism [6, 7].

Only a few studies formally analyzed associations between any of the key renal outcomes and clinical or biochemical features of chronic hypoparathyroidism. The most significant predictors for nephrocalcinosis in pediatric patients were degree of relative hypercalcemia and degree of hyperphosphatemia (*P* < 0.01) [10]. In pediatric patients, lower eGFR was associated with higher serum calcium concentrations and a greater proportion of time with relative hypercalcemia [10]. Similarly in adult patients, a significant inverse correlation was observed for eGFR levels with average time-weighted serum calcium and estimated proportion of time with hypercalcemia, as well as with age and disease duration (*P* < 0.001) [9]. In our clinical opinion, hypercalcemia in patients with chronic hypoparathyroidism is almost always attributable to overtreatment, making these factors exceedingly difficult to distinguish experimentally. Relatedly, although there is much debate in the medical community about the difference between ‘not adequately controlled’ and ‘not adequately treated’ with conventional therapy, this also cannot be answered on the basis of the published literature. No correlation was seen between the presence of kidney stones and serum calcium or 24-h urinary calcium excretion or with disease duration [21]. Similarly, there was no correlation between serum and urinary levels of calcium and the presence of the combined nephrolithiasis and/or nephrocalcinosis outcome [25].

Additional factors important to renal outcomes were identified in articles that did not undertake an association analysis but might be considered as surrogate markers or candidates for further exploration. In adult patients with chronic hypoparathyroidism, higher serum calcium-phosphate product values increased the risk of renal disease (ie, composite renal stones/eGFR <60 mL/min/1.73m^2^) [8]. The serum calcium-phosphate product level was within generally recommended reference ranges but was relatively high, leading Underbjerg et al. to suggest that treating physicians should target the lower part of the reference range. A similar point was made by the authors for target serum phosphate levels based on their association findings with increased risk for complications and mortality. Other studies in the general population reported that high serum phosphate was associated with harmful effects on renal function [32] and impairment of microvascular function in individuals with normal renal function [33]. In adult patients with chronic hypoparathyroidism, an increased number of hypercalcemic episodes and a longer duration of illness were associated with an increased risk of any incidence of renal disease [8]. In pediatric patients, a greater degree of hypocalcemia or repeated episodes of hypocalcemia were unexpectedly associated with the development of nephrocalcinosis [10], which could be explained by the need for higher doses of oral calcium to maintain normal serum calcium and/or concomitant hyperphosphatemia. However, these assertions require further investigation. The pediatric study provided definitive information about the presence of early mild renal impairment in children with chronic hypoparathyroidism. The authors noted that early renal impairment in childhood aligns with longitudinal studies in adults, and may progress to CKD in adulthood [12].

## Concluding remarks

Renal complications and an increased risk of adverse renal events in patients with chronic hypoparathyroidism who receive conventional therapy were observed consistently in a systematic literature review. There is an unmet need for additional large-scale studies, including more studies with standardized CKD definitions methodology, to better establish the factors that increase the risk of renal complications in patients with hypoparathyroidism.

## Data Availability

Not applicable.
